# Supplementary material for: An open-source framework for large-scale, flexible evaluation of biomedical text mining systems
Source: J Biomed Discov Collab. 2008 Jan 29;3:1. doi: 10.1186/1747-5333-3-1 (PMC2276192; doi:10.1186/1747-5333-3-1)
Supplement: Additional file 1 — Evaluation platform source code. The additional file contains the software evaluation framework discussed in this paper. The most current release can be downloaded from SourceForge.net [1]. See the accompanying README file for instructions on installation and use. Also included in the distribution is a collection of UIMA wrappers for some commonly used BioNLP tools and annotated corpora. [file 1747-5333-3-1-S1.zip › bionlp_uima_repository-1.0.1/docs/api/src-html/edu/uchsc/ccp/uima/ae/nlp/ei/gene/PennBioTagger_AE.html]

```
001    package edu.uchsc.ccp.uima.ae.nlp.ei.gene;
002    
003    import java.util.Iterator;
004    import java.util.List;
005    
006    import org.apache.uima.analysis_engine.ResultSpecification;
007    import org.apache.uima.analysis_engine.annotator.AnnotatorConfigurationException;
008    import org.apache.uima.analysis_engine.annotator.AnnotatorContext;
009    import org.apache.uima.analysis_engine.annotator.AnnotatorContextException;
010    import org.apache.uima.analysis_engine.annotator.AnnotatorInitializationException;
011    import org.apache.uima.analysis_engine.annotator.AnnotatorProcessException;
012    import org.apache.uima.analysis_engine.annotator.JTextAnnotator_ImplBase;
013    import org.apache.uima.jcas.JCas;
014    
015    import edu.uchsc.ccp.uima.annotation.CCPDocumentInformation;
016    import edu.uchsc.ccp.uima.util.UIMA_Util;
017    import edu.uchsc.ccp.util.external.penn.PennBioTaggerEISystem;
018    import edu.uchsc.ccp.util.nlp.annotation.TextAnnotation;
019    
020    public class PennBioTagger_AE extends JTextAnnotator_ImplBase {
021    
022        public static final String PARAM_TAGGER_MODEL_FILE = "pennBioTaggerModelFile";
023        
024        public static final String PARAM_TOKENIZER_MODEL_FILE = "pennBioTokenizerModelFile";
025        
026        public static final String PARAM_TAGGER_TYPE = "pennBioTaggerType";
027        
028        private PennBioTaggerEISystem pbtEIsys;
029        
030        public void initialize(AnnotatorContext ac) throws AnnotatorInitializationException, AnnotatorConfigurationException {
031    
032            String taggerModelFile;
033            String tokenizerModelFile;
034            String taggerType;
035            
036            try {
037                taggerModelFile = (String) ac.getConfigParameterValue(PARAM_TAGGER_MODEL_FILE);
038                tokenizerModelFile = (String) ac.getConfigParameterValue(PARAM_TOKENIZER_MODEL_FILE);
039                taggerType = (String) ac.getConfigParameterValue(PARAM_TAGGER_TYPE);
040            } catch (AnnotatorContextException ace) {
041                ace.printStackTrace();
042                taggerModelFile = null;
043                tokenizerModelFile = null;
044                taggerType = null;
045            }
046            
047            System.err.println("Initializing PENN BioTagger EI System ...");
048            pbtEIsys = new PennBioTaggerEISystem(taggerType, taggerModelFile, tokenizerModelFile);
049            
050            super.initialize(ac);
051        }
052        
053        
054        public void process(JCas jcas, ResultSpecification arg1) throws AnnotatorProcessException {
055            
056            /* get document text */
057            String docText = jcas.getDocumentText();
058    
059            Iterator docInfoIter = jcas.getJFSIndexRepository().getAnnotationIndex(CCPDocumentInformation.type).iterator();
060            String docID = "-1";
061            int documentCollectionID = -1;
062            if (docInfoIter.hasNext()) {
063                CCPDocumentInformation srcDocInfo = (CCPDocumentInformation) docInfoIter.next();
064                docID = srcDocInfo.getDocumentID();
065                documentCollectionID = srcDocInfo.getDocumentCollectionID();
066            }
067            
068            /* run the ei system */
069            List<TextAnnotation> entityAnnotations = pbtEIsys.getEntitiesFromString(docText, docID);
070            
071            for (TextAnnotation ta : entityAnnotations) {
072                ta.setDocumentCollectionID(documentCollectionID);
073                ta.setDocumentID(docID);
074            }
075            
076            /* add protein annotations to the CAS */
077            UIMA_Util uimaUtil = new UIMA_Util();
078            uimaUtil.putTextAnnotationsIntoJCas(jcas, entityAnnotations);
079        }
080    
081        
082    
083    }
```
